# Genome-Wide Libraries for Protozoan Pathogen Drug Target Screening Using Yeast Surface Display

**DOI:** 10.1021/acsinfecdis.2c00568

**Published:** 2023-04-21

**Authors:** Rhiannon Heslop, Mengjin Gao, Andressa Brito Lira, Tamara Sternlieb, Mira Loock, Sahil Rao Sanghi, Igor Cestari

**Affiliations:** † Institute of Parasitology, McGill University, Ste Anne de Bellevue, Montreal, QC H9X 3V9, Canada; ‡ Division of Experimental Medicine, McGill University, Montreal, QC H4A 3J1, Canada; § Faculté de Pharmacie de Tours, 31, Avenue Monge, 37200 Tours, France

**Keywords:** genomic library, yeast surface display, high-throughput sequencing, giardia, trypanosoma, metronidazole

## Abstract

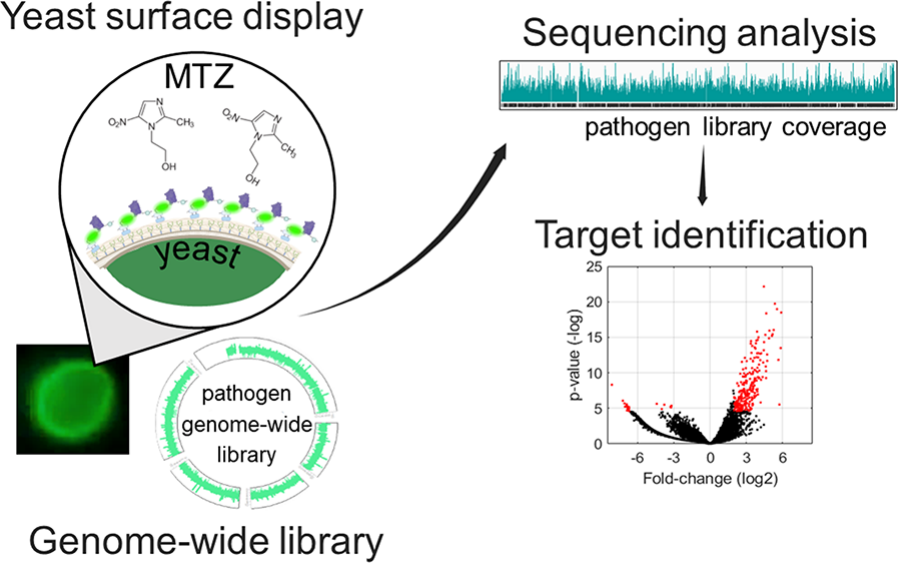

The lack of genetic tools to manipulate protozoan pathogens has limited the use of genome-wide approaches to identify drug or vaccine targets and understand these organisms’ biology. We have developed an efficient method to construct genome-wide libraries for yeast surface display (YSD) and developed a YSD fitness screen (YSD-FS) to identify drug targets. We show the efficacy of our method by generating genome-wide libraries for *Trypanosoma brucei*, *Trypanosoma cruzi*, and *Giardia lamblia* parasites. Each library has a diversity of ∼10^5^ to 10^6^ clones, representing ∼6- to 30-fold of the parasite’s genome. Nanopore sequencing confirmed the libraries’ genome coverage with multiple clones for each parasite gene. Western blot and imaging analysis confirmed surface expression of the *G. lamblia* library proteins in yeast. Using the YSD-FS assay, we identified bonafide interactors of metronidazole, a drug used to treat protozoan and bacterial infections. We also found enrichment in nucleotide-binding domain sequences associated with yeast increased fitness to metronidazole, indicating that this drug might target multiple enzymes containing nucleotide-binding domains. The libraries are valuable biological resources for discovering drug or vaccine targets, ligand receptors, protein–protein interactions, and pathogen–host interactions. The library assembly approach can be applied to other organisms or expression systems, and the YSD-FS assay might help identify new drug targets in protozoan pathogens.

Protein–ligand interactions are at the core of every cellular and molecular process in biology. They involve protein interactions with small molecules, nucleic acids, lipids, or other proteins. The discovery of protein–ligand interactions is essential for understanding signaling and developmental processes, gene regulation, pathogen–host interactions, and, notably, developing drugs and vaccines. However, discovering interacting partners is challenging and often relies on screening approaches, including yeast two-hybrid systems, overexpression systems, affinity chromatography coupled to mass spectrometry, or surface display technologies.

Yeast surface display (YSD) or phage display combined with high-throughput sequencing is one of the most successful approaches for protein–ligand discovery, mutational scanning, or protein engineering. Examples of their applications include the identification of glucocorticoid receptors,^1^ phage receptors,^2^ targets of drugs or inhibitors,^3^ vaccine targets,^4^ T-cell receptors,^5^ and antibody targets.^6,7^ YSD entails the expression of exogenous proteins encoded by DNA libraries on the surface of yeast cells. Each yeast cell expresses ∼10^5^ copies of a single protein on its surface, and thus, a large yeast population (∼10^8^) can easily represent a complete genomic library. The exogenous proteins are expressed in-frame with a surface anchoring system, e.g., N-terminal fused anchor proteins (SAG1, SED1), the a-agglutinin display system (Aga1p and Aga2p), or the Flo1p display system^8,9^ (Figure 1A). These anchored proteins are expressed on the exterior of the cell wall and expose the exogenous proteins for ligand interaction. While this approach has proven helpful, challenges in constructing genomic libraries have halted its broad application for routine protein–ligand discovery. Generating a eukaryote’s genome-wide library requires cloning 10^5^ to 10^7^ genome fragments into expression vectors, depending on fragment length and the organism’s genome size. DNA fragments are usually generated from genomic or complementary DNA (cDNA) via enzymatic digestion or random physical fragmentation and then cloned into expression vectors through ligation with restriction digested or T-tailed vectors.^10^ Sub-optimal library construction and expression systems can greatly diminish the efficiency of protein–ligand interaction screens. Inefficient library assembly can result in portions of the genome being absent or underrepresented in the library, thus limiting the scope and reliability of downstream screening applications.

**Figure 1:**
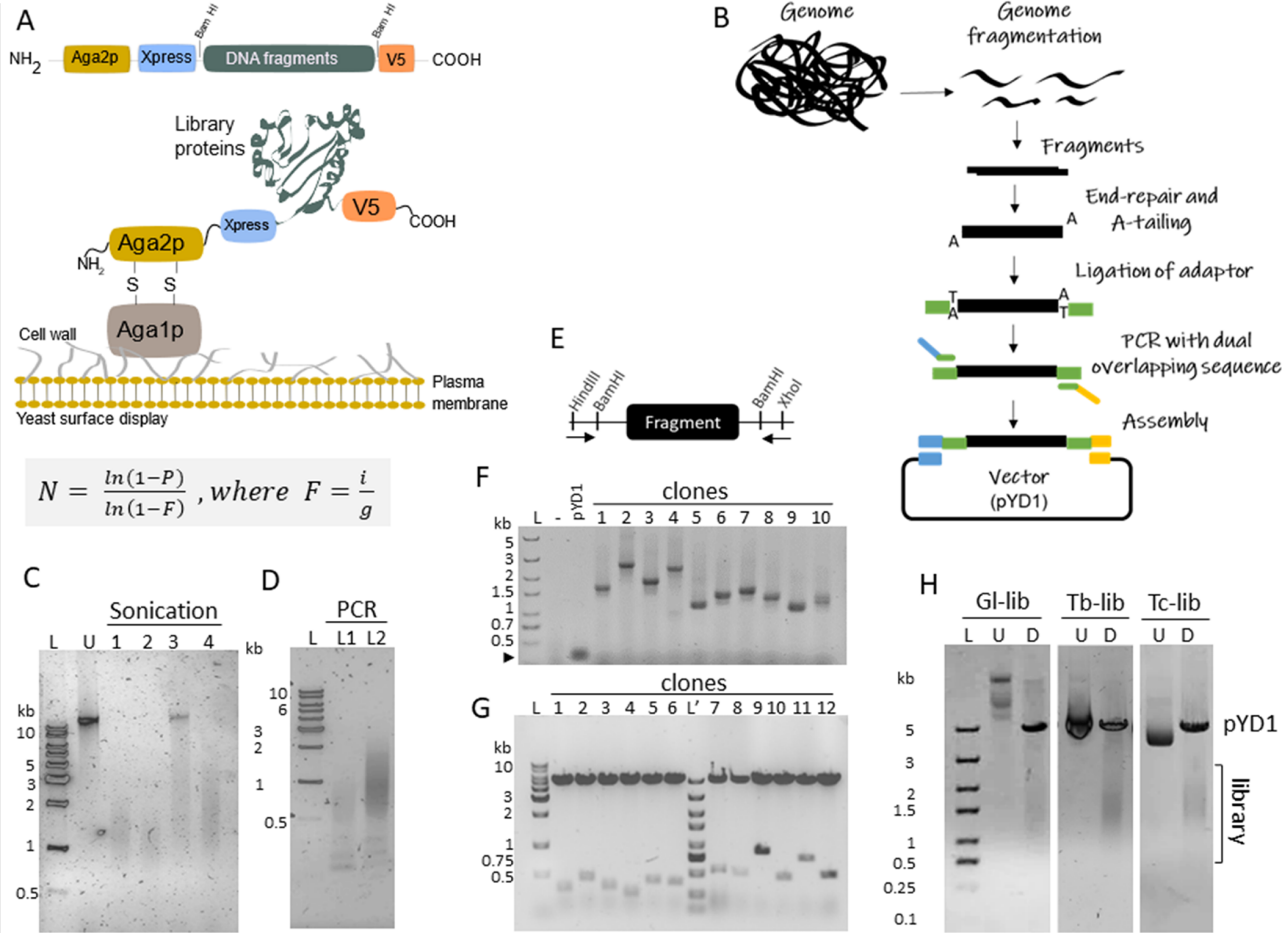
Assembly of genome-wide libraries for YSD. (A) Diagram of the pYD1 vector (top) showing library fragments cloned within Bam HI sites by Gibson assembly and (middle) yeast surface expression using the Aga1p-Aga2p system. (Bottom) Clarke and Carbon equation used to estimate the library size, i.e., the number of cloned fragments (*N*) given a probability (*P*). *F* is the quotient between insert fragment size (i) and genome size (g) in bp. (B) Diagram of the step-by-step genome-wide library preparation. (C) *G. lamblia* genomic DNA fragmentation by ultrafocused sonication. DNAs were not sonicated (U) or sonicated at (1) power (W) 25, duty factor (DF) 2%, 200 cycles per burst (cpb), for 45 s (s), or (2) 25 W, DF 10%, 100 cbp, 20 s; (3) 10 W, DF 2%, 700 cbp, 10s; and (4) 25 W, DF 10%, 200 cbp, 3 s. (D) PCR amplification of adapter-ligated library. Library adapter ligation with T4 DNA ligase (L1) or Blunt-TA ligase (L2). (E) Diagram of the pYD1 vector indicating Bam HI sites flanking cloned fragments and primers (arrows) used for fragment amplification. (F) PCR amplification of 10 *G. lamblia* library clones (from *E. coli* colonies) to validate the Gibson assembly step. The hyphen (-) indicates PCR reactions without colonies. (G) DNA restriction analysis of 12 plasmids from *T. cruzi* library clones with Bam HI enzyme to validate the Gibson assembly step. Clones are from two libraries with size-selected fragments of ∼300 bp (clones 1–6) and ∼700 bp (clones 7–12). (H) YSD libraries generated from *G. lamblia* (Gl-Lib), *T. brucei* (Tb-Lib), and *T. cruzi* (Tc-Lib) genomes. Library plasmids were digested with Hind III and Xho I (D). U, undigested library. L or L′ indicate DNA ladders (BioBasic).

The scarcity of genetic tools available to non-model organisms poses significant obstacles to developing genome-wide screenings. This is the case for many protozoan parasites, including kinetoplastids, diplomonads, and apicomplexan, responsible for huge health and economic burden worldwide.^11^*Trypanosoma cruzi*, the causative agent of Chagas disease (ChD), affects ∼8 million people in South and Central America. ChD has spread to Europe, Australia, and Japan and affects over 300,000 people in the United States.^11^*Trypanosoma brucei* causes African Trypanosomiasis, also known as Sleeping Sickness, and affects humans and animals with significant health and agricultural impact in Sub-Saharan Africa.^11^*Giardia lamblia* (also known as *G. intestinalis*) causes intestinal diseases with nearly 200 million yearly cases, especially among children and immunocompromised people in low and middle-income countries.^12^ There are no vaccines against these diseases, and the available drugs have limited efficacy and severe side effects.^11^ While genetic approaches have contributed to understanding *T. brucei* biology and drug target discovery,^13−15^ much less has been done for other protozoan pathogens, in which genetic manipulation remains a challenge. Hence, the development of parasite genome-wide libraries for YSD may help advance the knowledge of these organisms’ biology and the development of therapeutics, including identifying drug and vaccine targets.

We have developed an efficient approach to construct genome-wide libraries for YSD. Here, we show the usefulness of our method by generating genome-wide libraries for *T. brucei*, *T. cruzi*, *andG. lamblia* encoding polypeptides for yeast surface display. The diversity of the libraries ranges from ∼2 × 10^5^ to 4 × 10^6^ clones, representing ∼6- to 30-fold coverage of the parasite genome. Nanopore sequencing confirmed the libraries’ genome coverage, and computational analysis predicted the libraries to encode polypeptides in the protein domain range (20–200 amino acids, aa), which is ideal for identifying ligand-binding regions or epitopes targeted by antibodies. As a proof-of-concept, we transformed yeast with the *G. lamblia* library and confirmed its polypeptide expression. We developed a YSD fitness screen (YSD-FS) assay for drug target discovery. Using the YSD-FS assay, we identified known interactors of metronidazole, a drug used to treat giardiasis, and new candidate interacting proteins. Notably, the data revealed enrichment in nucleotide-binding domain sequences conferring yeast increased fitness to metronidazole, implying that metronidazole might target multiple proteins containing nucleotide-binding domains. The libraries are valuable biological resources for discovering drug or vaccine targets, ligand receptors, and protein–protein interactions and investigating pathogen–host interactions. The library assembly method can be applied to other organisms or expression systems, and the YSD-FS assay will pave the way to identifying new drug targets in protozoan pathogens.

## Results

### Efficient Construction of Genome-Wide Libraries for YSD

To generate genome-wide libraries for YSD, we devised an approach to clone parasite genomic fragments into the pYD1 vector (Figure 1A,B). The method entails genome fragmentation followed by fragments end-repairing and A-tailing for adapter ligation. The adapter-ligated fragments are amplified by PCR with primers that pair with adapter sequences and have ∼20 bp vector overlapping sequences used for fragment cloning by Gibson assembly (Figure 1B). Gibson assembly is a method for joining DNA molecules using a single isothermal reaction and requires an ∼20 bp overlap region between joining molecules,^16^ i.e., fragments and vectors. In this approach, a 5′-exonuclease generates 3′-overhangs in the joining molecules, which are then annealed. A DNA polymerase fills gaps between annealed strands, and a DNA ligase seals the nicks in the strands. We initially selected the protozoa *G. lamblia* for genome-wide library construction because it has a haploid genome of 12.1 Mb,^17^ thus smaller than most eukaryotes. This organism’s genome is also primarily organized in arrays of exons with an average gene size of ∼1.5 kb and a coding density of 81.5% of the genome.^17,18^ The giardia genomic DNA was fragmented in sizes ranging ∼0.5–2 kb by ultrafocused sonication (Figure 1C) to generate a YSD library encoding polypeptides. The genome fragments were end-repaired and A-tailed followed by ligation of a nanopore forked adapter, which was verified by PCR library amplification (Figure 1B,D). Amplicons from a 16-cycle PCR reaction were used for Gibson assembly reactions followed by bacterial transformation. PCR analysis of randomly selected clones from transformed bacteria confirmed that library clones contained fragments ranging between about 0.5 and 2 kb (Figure 1E,F), consistent with the fragmentation, and Sanger sequencing of isolated plasmids confirmed that the sequences originated from the parasite genome. Using the Carbon and Clarke equation^19^ (Figure 1A), we calculated that 36,880 cloned fragments were required to have each *G. lamblia* genome fragment represented in the library with a 99% probability. After library assembly, we obtained ∼2.0 × 10^5^ clones, which represents 5.3-fold the necessary calculated number of clones with an estimated density of 31 clones per gene (Table 1). Restriction analysis of the constructed library indicated fragments ranging between about 0.5 and 2 kb (Figure 1H), consistent with fragment size selection.

**Table 1:** Characteristics and Diversity of *T. cruzi* (Tc-Lib), *T. brucei* (Tb-Lib), and *G. lamblia* (Gl-Lib) YSD Libraries

library features	Tc-Lib	Tb-Lib	Gl-Lib
library sequence mean length^a^	1057 bp	644 bp	860 bp
total number of clones	4.0 × 10^6^	7.2 × 10^5^	1.9 × 10^5^
library genome coverage^b^	29.6-fold	6.7-fold	5.3-fold
number of clones per gene^c^	270	41	31
gene enrichment	100%	100%	100%
predicted number of polypeptides^d^	248,946	184,876	48,926
predicted polypeptide range (aa)^e^	13–559	13–275	13–488
number of predicted polypeptides per gene^c^	18.9	18.7	8.6

a Library sequence mean length calculated from nanopore sequencing data.

b Fold change of the total number of clones obtained compared to the number of clones necessary for one genome coverage at a probability of 99% using the Clark and Carbon equation.^49^

c Library size corrected by percent of coding genome.

d Predicted number of proteins from fastq sequences using the Libframe tool corrected by library size.

e Polypeptide length predicted using the Libframe tool.^26^

**Figure 2:**
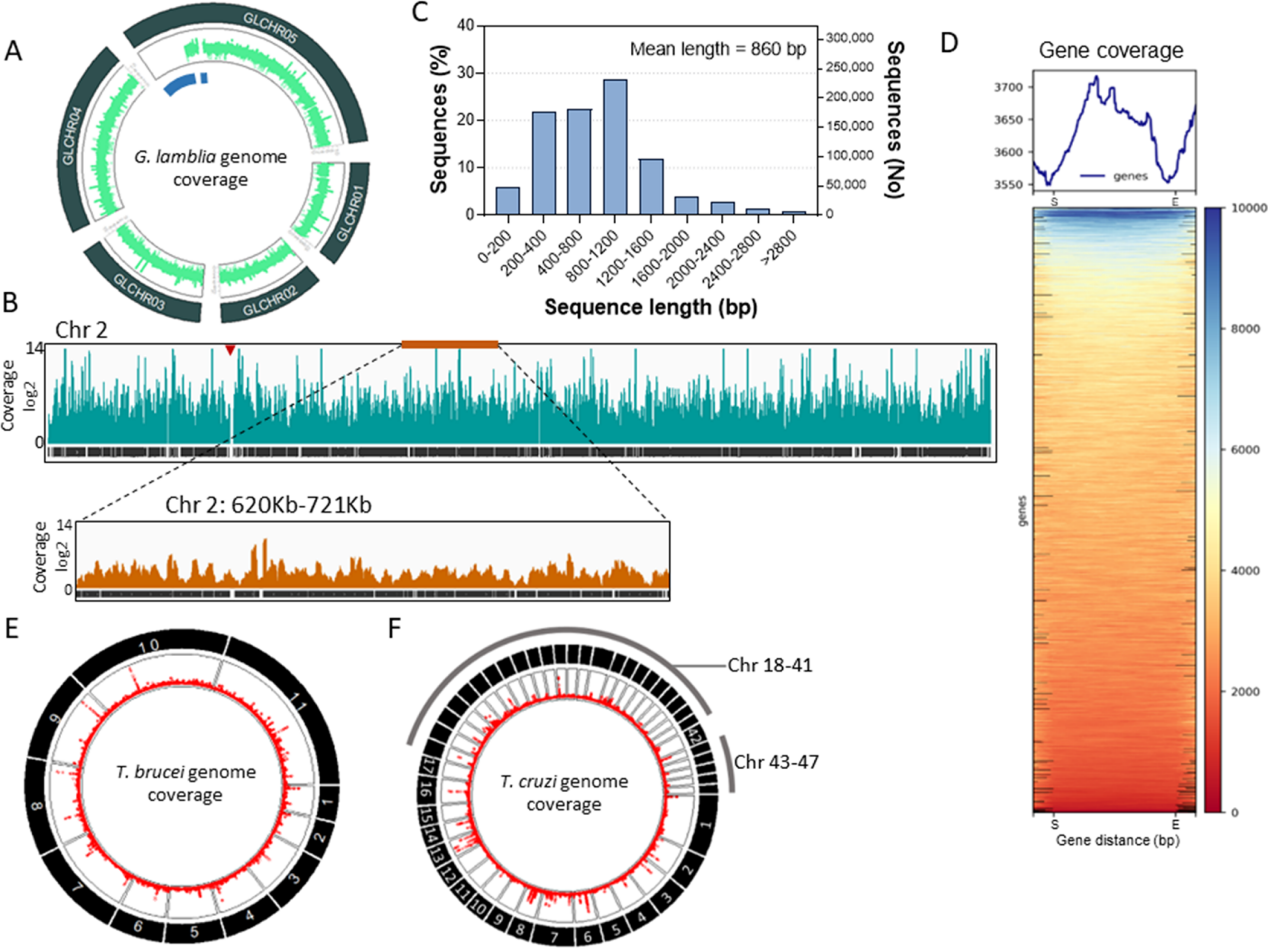
Nanopore sequencing analysis of YSD libraries. (A) Circular plot shows the mapping of *G. lamblia* library nanopore sequencing (light green) to all five chromosomes (dark green rectangles). The blue segments indicate the lack of sequence in the reference genome data (hence no mapping). (B) Analysis of nanopore read coverage of chromosome 2 (Chr 2). A segment of ∼100 Kb of Chr 2 (orange bar) is highlighted below. The red arrowhead indicates a lack of sequence in the reference genome. (C) Distribution of *G. lamblia* genome fragment sequence lengths from nanopore sequencing data. A total of 806,545 reads were used for analysis. No, number. (D) Heatmap of all *G. lamblia* genes shows gene read coverage. All gene sequences were resized to 2 kb for read coverage analysis. S, start codon, E, stop codon. A 0.5 Kb upstream the start codon and downstream the stop codon are shown. (E, F) Circular plot shows the mapping of nanopore sequencing (red lines) of *T. brucei* (E) and *T. cruzi* (F) libraries to the corresponding parasite genome chromosomes (black rectangles).

To determine the library coverage of the genome, we sequenced the library using Oxford nanopore sequencing. The long-read technology generates sequences covering the complete DNA sequence of the cloned fragments. Nanopore sequencing of the *G. lamblia* library (Gl-Lib) resulted in ∼90% of reads mapped to the *G. lamblia* WB strain reference genome chromosomes and resulted in 27.3× read coverage of the genome (Table 2, Figure 2A). Inspection of the parasite chromosomes (chr) showed extensive library representation of the genome with nearly no gap in coverage (Figure 2A,B). The apparent gaps observed in chr 5 relate to the lack of a defined sequence in the reference genome (represented by Ns in the reference genome). About 60% of the library included fragments ranging between 0.4 and 1.6 kb with an average size of 860 bp (Figure 2C, Table 1). The library read lengths correlated with library fragmentation size, but it also indicated a slight bias toward DNA fragments of small size (Figure 2C, Table 1). Moreover, it also indicated that the library contains fragments to encode predominantly protein motifs or domains^20−22^ compared to full-length proteins. The results also revealed that all *G. lamblia* annotated genes were represented in the library (Table 1), and essentially, all gene sequences were represented by library fragments (Figure 2D). The data show that the method is efficient for constructing genome-wide libraries.

**Table 2:** Nanopore Sequencing Metrics of *T. cruzi*, *T. brucei*, and *G. lamblia* YSD Libraries

library features	Tc-Lib	Tb-Lib	Gl-Lib
total sequenced bases	8.74 Gb	3.56 Gb	1.97 Gb
mean sequencing quality^a^	12.5	11.7	12.1
total number of reads	8,267,306	4,618,344	806,545
sequence genome coverage	146.9×	131.9×	27.3×

a Q-score.

### Constructing Libraries of Organisms with Large Genomes

To further validate our approach, we generated libraries for *T. brucei* and *T. cruzi*, with haploid genomes being 35 Mb and 44 Mb, respectively. These parasite genomes are about 3 to 4 times larger than the *G. lamblia* genome and thus helped test the method’s efficiency and evaluate potential bias associated with genome size. Trypanosome genomes are organized primarily in segments of exons with genes averaging a size of ∼1.5 kb. After library assembly and transformation, we obtained 7.2 × 10^5^ and 4.0 × 10^6^ clones for *T. brucei* and *T. cruzi*, respectively (Table 1), representing 6.7- and 29.6-fold the calculated coverage of their genomes at 99% probability, and estimated 41 and 270 clones per gene (Table 1). Restriction enzyme analysis confirmed the library fragment ranging between about 0.5 and 2 Kb (Figure 1G,H). After constructing multiple genome-wide libraries, we found that optimizing the Gibson assembly reaction, which involves defining an optimal ratio between DNA fragments and a plasmid vector, is a critical and often necessary step in library construction. After optimization of the Gibson assembly step, we consistently obtained 100% efficiency in library generation using this method. The libraries are hereafter named Tc-Lib and Tb-Lib, respectively, for *T. cruzi* and *T. brucei* libraries.

The nanopore sequencing of Tb-Lib and Tc-Lib resulted in 131.9× and 186.1× genome coverage, respectively (Table 2). Both Tb-Lib and Tc-Lib showed a thorough representation of their respective organism genomes covering all chromosomes (Table 1, Figure 2E,F). The sequencing analysis also revealed an average fragment size of ∼0.7 to 1 kb for the libraries, and every single gene of the parasite’s genome was represented in the libraries (Table 1). There was an apparent enrichment bias for large gene families in all libraries, which is observed as genome coverage peaks in the circular plots (Figure 2A,E,F, Figure S1). The result is unlikely related to sequence selection during library construction, but it may reflect the poor genome assembly of repetitive regions, especially segments containing large gene families such as mucins or mucin-associated proteins (MASPs) in *T. cruzi*, variant surface glycoproteins (VSGs) in *T. brucei*, and variant surface proteins (VSPs) in *G. lamblia*. The library fragments also covered untranslated regions of the genome. The effective assembly of the three organism’s libraries implies that genome size is not a constraint for library construction by this method, and the data show that the libraries are diverse and comprehensive.

### Prediction of Library Polypeptides and Expression in Yeast for Surface Display

**Figure 3:**
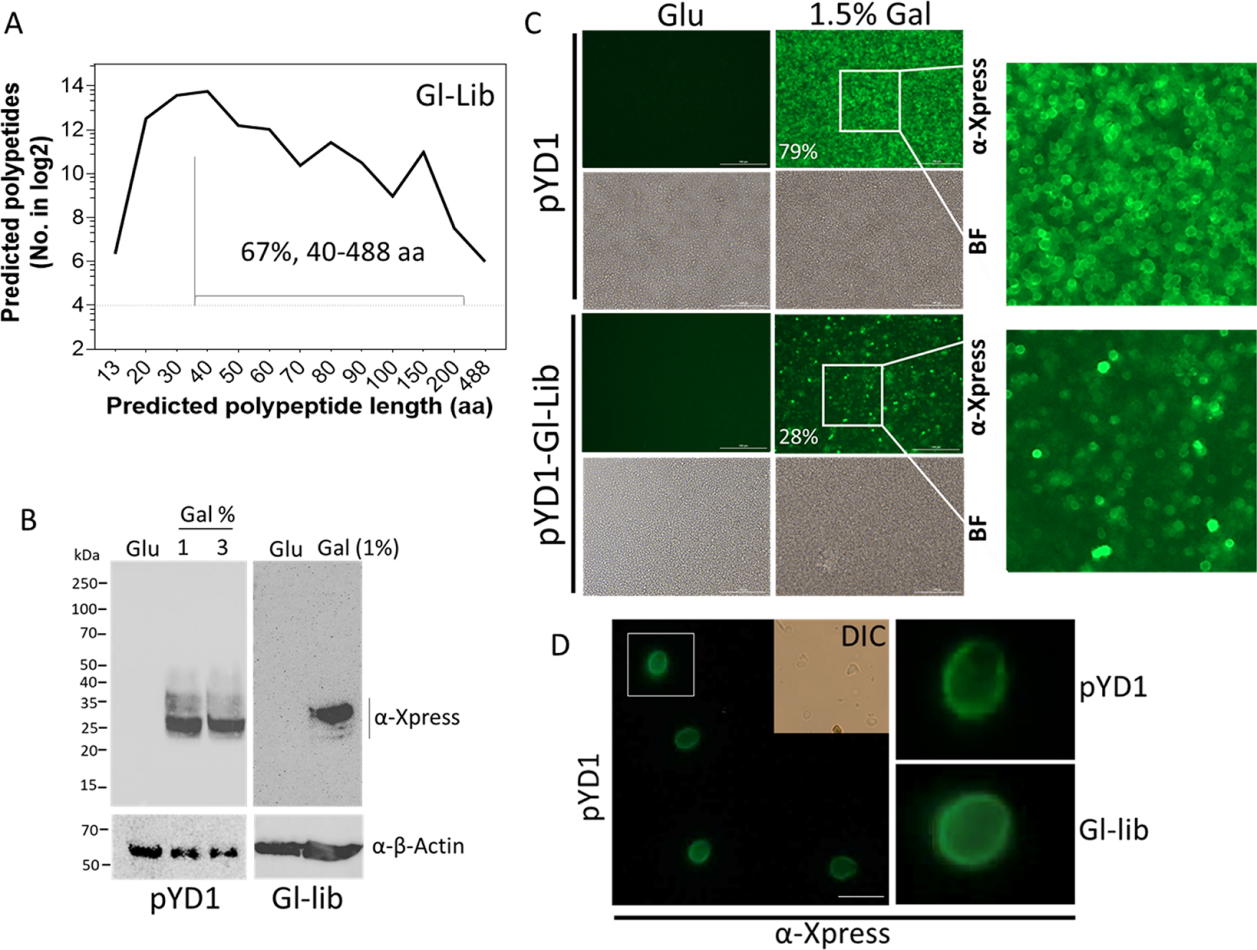
Expression analysis of Gl-Lib YSD. (A) Length distribution analysis of Gl-Lib predicted unique polypeptides expressed in-frame with Aga2p using the Libframe tool^26^ (https://github.com/cestari-lab/Libframe-tool). (B) Western blot analysis of yeast EBY100 strain transformed with pYD1 (left) or Gl-Lib (right). Proteins were resolved in 12% SDS/PAGE, transferred to a nitrocellulose membrane, and blotted with anti-Xpress antibodies. Membranes were stripped and reblotted with anti-β-actin antibodies. Glu, non-induced (2% glucose); Gal, induced with 1–3% galactose in medium containing 2% raffinose. (C) Image reader analysis of pYD1 or Gl-Lib transformed yeast. Image analyzed with a Cytation 5 image reader in 96-well plates. Bar, 100 μm. Cells were stained with mouse anti-Xpress and AlexaFluor-488-labeled anti-mouse IgG (green). Quantification of Xpress positive cells by flow cytometry (1% Gal) is indicated in % (Figure S2). Glu, 2% glucose; Gal, galactose. (D) Fluorescence microscopy analysis of yeast expressing pYD1 or Gl-Lib induced with 1.5% galactose. Cells were treated as described in (C). BF, color bright field. DIC, Differential interference contrast. Bar, 10 μm. Images comparing pYD1 and Gl-Lib from (C) or (D) were taken with the same light exposure and acquisition time.

We developed a computational tool that predicts the library’s polypeptide sequences potentially expressed on the yeast surface. The Libframe tool uses the fastq data from nanopore sequencing. It identifies library sequences in frame with the sequences of interest, here, the Xpress epitope downstream from the Aga2p coding sequence (Figure 1A). Then, it translates in-frame DNA sequences outputting the predicted amino acid sequences and polypeptide length. Analysis of a pool of library sequences indicated 10^4^ to 10^5^ polypeptides ranging 13–488 amino acids (aa), 13–275 aa, and 13–559 aa for the Gl-Lib, Tb-Lib, and Tc-Lib, respectively (Figure 3A, Table 1). Further analysis of the Gl-Lib indicated that 33% of the in-frame library DNA sequences generated predicted polypeptides ranging from 13 to 39 aa, whereas 67% of sequences encoded proteins ranging from 40 to 488 aa, which corresponds to about 16,146 and 32,780 predicted polypeptides, respectively, and thus a calculated average of 8.6 polypeptides per gene (Table 1). Notably, over 90% of protein domains range between 20 and 200 aa with an average of ∼60 aa, whereas motifs and ligand binding sites are 7–8 aa and ∼18 aa, respectively,^20−23^ implying that the libraries can generate polypeptides encompassing protein functional groups. The library polypeptide range reflects the size distribution of cloned genomic fragments (Figure 2C, Table 1) and early stop codons or frameshifts between library sequences and Aga2p. Early stop codons result from DNA fragments originating from untranslated regions of the genome (18.5% of the *G. lamblia* genome) and coding sequences cloned out of frame, which is common in genomic libraries. Importantly, the large library sizes with hundred thousand to millions of cloned fragments ensure that polypeptides of coding sequences with sufficient length to cover functional protein sequences, motifs, and protein domains are included.

We transformed the *Saccharomyces cerevisiae* EBY100 strain with the Gl-Lib or the pYD1 vector to validate library expression. To represent a library diversity of ∼10^6^ clones, high-efficiency yeast transformation is required to obtain at least 10-fold of the library size. Thus, we optimized library transfection in yeast by electroporation and obtained a transformation efficiency of ∼10^7^ cfu/μg of library DNA, which resulted in transformed yeast representing ∼364 times the Gl-Lib size (Table S1). The pYD1 vector has an N-terminal Xpress epitope in frame with Aga2p, which is expressed fused to the library sequences under control of a promoter induced by galactose (gal) or repressed by glucose (glu) (Figure 1A). Western analysis of gal-induced Gl-Lib confirmed the expression of fragments from ∼25 to 35 kDa (Figure 3B). The size range correlates with our computational prediction of expressed polypeptides as most of library fragments are predicted to generate 2–7 KDa polypeptides, and the predicted molecular weight of Aga2p plus the Xpress epitope is ∼24 kDa.^24,25^ We analyzed the expression of the Gl-Lib using a Cytation 5 cell image reader using anti-Xpress antibodies (Figure 3C). The data showed a uniform detection of surface Xpress from pYD1 transformed yeast, and flow cytometry quantification showed that ∼79% of the yeast uniformly expressed high levels of Xpress epitope (Figure S2). In contrast, the Gl-Lib expressing yeast showed a heterogeneous detection of Xpress (∼28% positive by flow cytometry), indicating a mixed population of high- and low-expressing cells (Figure S2). The low percentage of Xpress positive cells in the Gl-Lib likely reflects interference of the library proteins with anti-Xpress antibodies binding to the adjacent Xpress epitope. The data is consistent with other YSD libraries generated using the Aga1p-Aga2p expression system.^10^ Microscopy analysis further confirmed the surface Xpress exposure, indicating a functional yeast surface display system (Figure 3D). The data show that our methodology generates diverse and functional genome-wide YSD libraries.

### Screening for *G. lamblia* Proteins That Interact with Metronidazole

**Figure 4:**
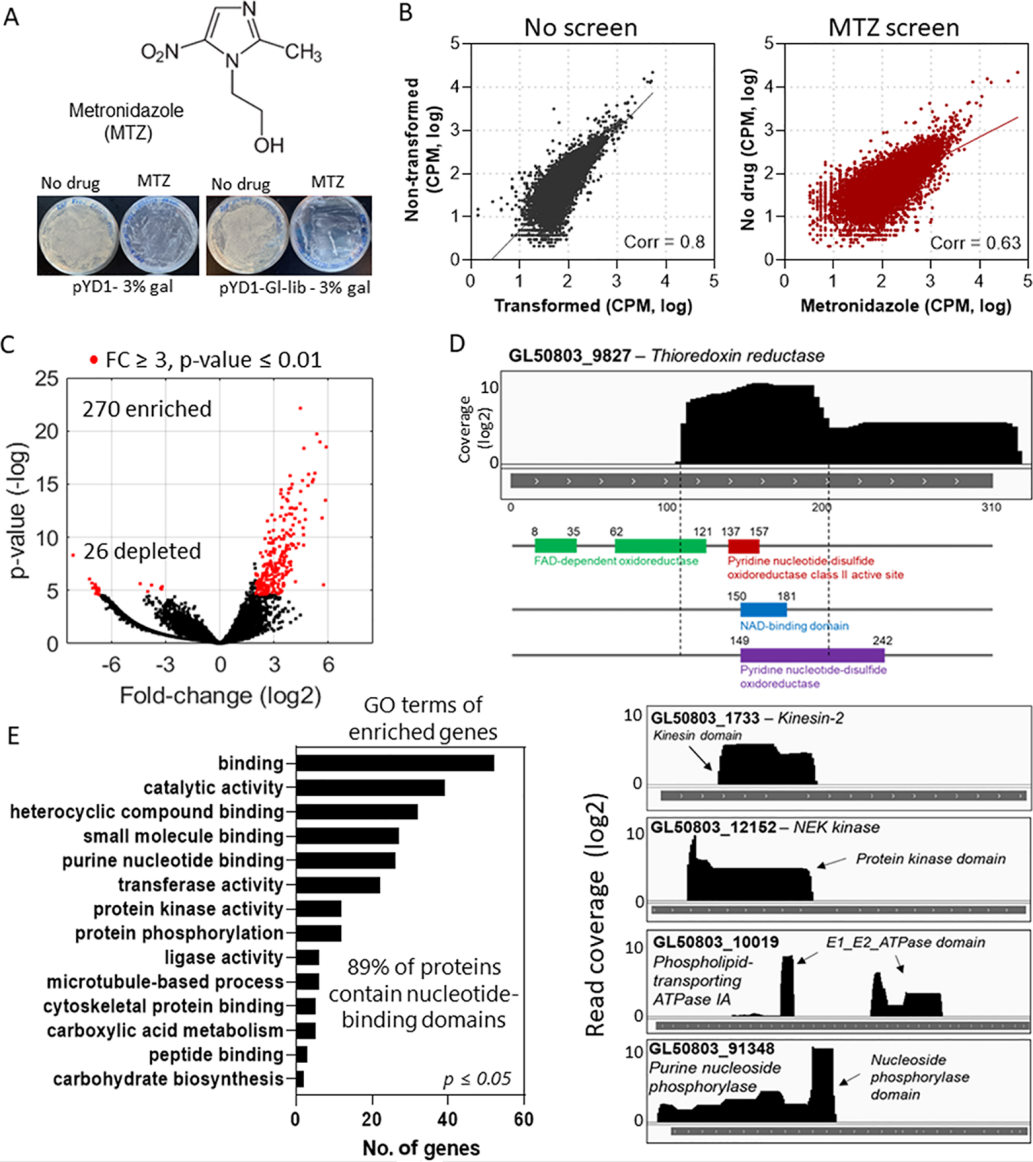
YSD-FS to identify targets of metronidazole. (A) Diagram of the drug metronidazole (top). Pictures of Petri dishes with yeast expressing the pYD1 (empty vector) or pYD1-Gl-Lib. Libraries were induced with 3% galactose in the absence or presence of 20 mM of metronidazole. (B) Scatter plot of nanopore read counts per million (CPM) of yeast transformed library compared with non-transformed library (left, No screen) and yeast expressing Gl-Lib in the absence or presence of metronidazole (right, MTZ screen). The Pearson coefficient of correlation (Corr) comparing datasets is shown. (C) Volcano plot shows genes enriched or depleted after YSD-FS with metronidazole. FC, fold-change. Statistically significant enriched or depleted hits are indicated in red. (D) Read coverage analysis shows the location of library fragments mapped to genes enriched in the metronidazole screen. The coverage was calculated by comparing metronidazole-treated vs non-treated samples. Top, the diagram of thioredoxin reductase shows read coverage mapping nucleotide-binding domains. Bottom, read coverage over nucleotide-binding domains in different proteins. (E) Go enrichment analysis of the annotated genes enriched in the YSD-FS with metronidazole.

We posited that YSD libraries are useful tools for drug target screenings. The yeast surface expression exposes library proteins to small ligands outside the cell, favoring ligand–protein interactions. The interaction of library proteins with drugs may lead to drug depletion, drug enzymatic inactivation, or drug activation into toxic derived molecules, all of which may affect the growth of interacting yeast clones in the population. Hence, we performed a YSD-FS by growing yeast in the presence or absence of metronidazole to identify clones showing altered fitness in the presence of the drug. Gl-Lib expressing yeasts were grown for three days in vehicle only or in the presence of metronidazole, and the grown yeast population was submitted to four rounds of fitness selection in metronidazole. The library plasmid DNAs were recovered from yeasts for fragment DNA sequencing using Oxford nanopore technology. Sequencing analysis of the vehicle-treated YSD showed a 0.8 Pearson correlation with the non-transformed (original) library, implying only a slight deviation in the yeast-expressed library from the generated library (Figure 4B, see No screen). However, drug-treated Gl-Lib expressing yeast showed a low correlation with the vehicle-treated library (Pearson correlation of 0.63), implying that drug treatment altered the fitness of the yeast population expressing the Gl-Lib (Figure 4B, MTZ screen). Similar results were obtained by comparing the drug-treated Gl-lib yeast library to the non-transformed library (Pearson correlation of 0.65) (Figure S3). Analysis of metronidazole treated vs vehicle-treated Gl-Lib expressing yeast revealed 270 genes enriched and 26 depleted (Figure 4C, fold-change ≥3, *p*-value ≤0.01). Of these genes, 108 genes (96 enriched and 12 depleted) had annotations other than hypothetical proteins and included bonafide interactors of metronidazole such as thioredoxin reductase, purine nucleoside phosphorylase, and a putative ortholog of metronidazole target protein 1.^27^ The screen also identified 13 kinases, primarily from the expanded NEK kinase family; 14 protein 21.1 (Ankyring domain-containing proteins), and genes involved in DNA metabolism, antioxidant metabolism, and membrane transporters (Table 3 and Table S2). Importantly, analysis of the enriched library fragments revealed specific gene sequences associated with yeast increased fitness to metronidazole (Figure 4D). The sequences matched primarily to nucleotide binding regions, e.g., nicotinamide adenine dinucleotide (NAD)-binding domains, P-loop containing nucleotide-binding (NTPase) domain, or ATPase domains, indicating that metronidazole may interact with different proteins containing nucleotide-binding domains, i.e., ATP-binding domains or nucleotide cofactor domains (Figure 4D). Gene ontology (GO) analysis of the annotated genes showed enrichment in heterocyclic compound binding, small molecule binding, purine nucleotide binding, transferase, and protein kinase activities (Figure 4E), confirming a bias toward nucleotide-binding proteins in the genes identified. Analysis of the protein domains encoded by the identified genes revealed that 89% had an annotated nucleotide-binding domain (Figure 4E,F). Hence, the screen identified proteins known to interact with metronidazole, potential drug-binding domains, and new candidate proteins to help identify metronidazole targets and mechanisms of action. The data also shows that YSD-FS is a useful approach for drug target screening without the chemical labeling or modification of drugs.

**Table 3:** Selection of Top *G. lamblia* Enriched or Depleted Genes from Metronidazole YSD-FS^a^

No	gene ID	log FC	*p-*value	description
1	GL50803_4563	5.895305	9.35 × 10^–9^	copine I
2	GL50803_92729	5.380395	2.73 × 10^–9^	fatty acid elongase 1
3	GL50803_8916	4.620139	1.85 × 10^–5^	signal recognition particle 68 kDa
4	GL50803_115047	4.588984	2.19 × 10^–7^	VSP
5	GL50803_10019	4.464974	2.42 × 10^–10^	phospholipid-transporting ATPase IA, putative
6	GL50803_16841	4.175765	3.09 × 10^–6^	DinF protein
7	GL50803_13982	4.160367	7.63 × 10^–6^	protein 21.1
8	GL50803_7030	4.083904	3.34 × 10^–5^	prefoldin subunit 3, putative
9	GL50803_12157	4.031597	9.64 × 10^–5^	RabB
10	GL50803_101074	3.991449	2.85 × 10^–6^	VSP with INR
11	GL50803_9827	3.980817	8.27 × 10^–5^	thioredoxin reductase
12	GL50803_6624	3.897405	1.39 × 10^–7^	kinase (putative MTP1)
13	GL50803_19575	3.83776	4.85 × 10^–7^	variant-specific surface protein
14	GL50803_16774	3.711709	1.27 × 10^–6^	trichohyalin
15	GL50803_28666	3.686641	0.000269	diphthine synthase
16	GL50803_10516	3.675562	0.001123	leucine carboxyl methyltransferase
17	GL50803_9808	3.668521	5.53 × 10^–6^	chaperone protein DnaJ
18	GL50803_9079	3.577647	0.000399	ERP2
19	GL50803_10573	3.558475	8.34 × 10^–6^	protein 21.1
20	GL50803_17333	3.543379	5.27 × 10^–5^	kinesin-2
21	GL50803_137681	3.446379	0.000622	VSP
22	GL50803_114192	3.442315	2.71 × 10^–6^	kinase, NEK
23	GL50803_6101	3.419173	0.000551	variant-specific surface protein
24	GL50803_94440	3.399418	5.19 × 10^–7^	dynein heavy chain
25	GL50803_12152	3.319794	0.000107	kinase, NEK
26	GL50803_6055	3.117717	0.008258	FtsJ-like protein
27	GL50803_4629	3.108928	0.001777	BC2-ORF3
28	GL50803_137740	–8.12615	0.000244	VSP
29	GL50803_16520	–7.21418	0.002303	Sec24-like
30	GL50803_35428	–6.90545	0.004618	valine-tRNA ligase
31	GL50803_7653	–6.88015	0.00508	kinase, NEK-like
32	GL50803_15972	–6.8742	0.00459	protein 21.1
33	GL50803_95653	–6.83489	0.006444	coiled-coil protein
34	GL50803_96670	–6.82082	0.005544	potassium-transporting ATPase alpha chain 1
35	GL50803_114535	–6.80025	0.007135	kinase, NEK
36	GL50803_17023	–6.77564	0.005418	protein 21.1
37	GL50803_5603	–6.70283	0.009188	coatomer gamma subunit
38	GL50803_12057	–6.69677	0.006558	gamma tubulin ring complex
39	GL50803_17465	–3.26899	0.005458	SMC4-like protein
40	GL50803_112801	–3.22903	0.006021	VSP

a Only a selection of 27 enriched and 13 depleted genes with fold-change (FC) ≥ 3 logs and *p-*value ≤ 0.01 is shown. For a complete list, see Table S2.

## Discussion

We have developed a method to generate genome-wide libraries and demonstrated the method’s efficiency by constructing three genome-wide libraries of protozoan pathogens. We have optimized the libraries’ expression in yeast for surface display and developed an assay combining YSD-FS and high-throughput sequencing to identify potential drug-interacting proteins, demonstrated by using the *G. lamblia* YSD library to screen for potential metronidazole interacting proteins. The screen identified known metronidazole binding proteins, corroborating the assay strength and new potential metronidazole binding targets. The assay also revealed specific protein domains associated with yeast-altered fitness to metronidazole, indicating potential metronidazole binding sites. The libraries, screening datasets, and methods are valuable resources for identifying drug targets, especially for organisms with limited genetic tools.

Generating eukaryote genomic libraries requires cloning between 10^5^ and 10^6^ fragments, which can cause a bottleneck in library construction. To address this problem, we used Gibson assembly to join DNA fragments,^16^ which also facilitates library transfer between different vectors. In our approach, we ligated a forked adapter to the genomic fragments and then used it for fragment amplification with primers containing overlapping sequences to the vector. The ligation of the forked adaptor is straightforward and is routinely used by many laboratories to generate DNA or RNA sequencing libraries. We opted for an Oxford nanopore T/A-based forked adapter because it facilitates the preparation of long-read DNA sequencing of the libraries. In this approach, a single PCR step generates barcoded fragments for DNA sequencing, avoiding long protocols associated with nanopore sequencing library preparation. Still, we anticipate that other adapters, including Illumina or customized adapters, can be used to replace the nanopore adapters during library design. The ∼20 bp overlapping sequence matching the receptor vector (e.g., pYD1) can also be replaced by any vector’s sequence, which provides library transfer flexibility for multiple applications. The timeframe to generate a eukaryote genomic library using this method is about two to three weeks. Other methodologies for library generation include DNA cloning based on ligation of T-tailed vectors or restricted digested DNAs. One limitation of DNA or cDNA restriction enzyme digestion and ligation results from bias in DNA digestion depending on the distribution of restriction enzyme sites in the DNA. At earlier stages of this work, we also attempted generating libraries by DNA ligation using T/A cloning vectors. Although efficient for cloning, custom-made T/A-cloning vectors are not as efficient for large-scale library construction, and they also limit library transfer among different vectors.

We generated three genome-wide libraries with protozoan genomes varying between 12.1 and 44 Mb. There was no bias in library construction related to genome size, and libraries were diverse and complete. Still, a minor tendency toward cloning small fragments was observed, which is common to any cloning approach because small DNA fragments can be overly represented in DNA samples compared to large DNA sequences. The libraries’ high genome coverage and diversity increase the chance that clones containing the desired coding region in frame with Aga2p for yeast surface expression are included. Computational analysis based on nanopore reads predicted thousands of polypeptides in frame with Aga2p. The predicted amino acid sequences included polypeptides in the length range of protein motifs, domains,^20−22^ and potential epitopes for protein–ligand binding, including the discovery of antibody targets, protein–domain interaction analysis, and small molecule target screens. We also optimized a protocol for the high-efficiency transformation of yeast and found that the transformed yeast cells preserved the library’s completeness and diversity. We anticipate that the method can help assemble libraries for different applications, including the expression of full-length cDNA sequences, stage-specific transcripts, or gene-specific mutagenesis libraries.

The three libraries generated and validated via nanopore sequencing for *G. lamblia*, *T. brucei*, and *T. cruzi* are powerful resources for exploring basic and translational biology. Advances in *T. brucei* genetic tools, including RNA interference and genetic screens, have helped identify drug targets and advance our understanding of parasite biology.^13,15,28^ However, much less has been done for *T. cruzi* and *G. lamblia*, which have undeveloped genetic tools. RNA interference is present in *G. lamblia*(29) but not in *T. cruzi*,^30^ and CRISPR/Cas9 has been used in *T. cruzi*.^31,32^ However, applying those tools has challenges and limitations, including variability in RNA interference knockdown efficiency, poor parasite transfection efficiency, and lack of regulatable gene expression systems, limiting scaling the approaches for high throughput applications. To date, not a single genetic screen has been performed in *T. cruzi* or *G. lamblia*, which attests to the challenges in the genetic manipulations of these organisms. Hence, harnessing yeast genetics for surface display will help to explore the parasite genomes for drug and vaccine target discovery and advance our knowledge of these organisms’ basic biology.

The YSD technology presents several advantages compared to other protein overexpression systems. Yeast has a cell wall that can limit cell permeability to extracellular ligands and thus prevents potential ligand interactions with intracellular protein targets. Moreover, yeast metabolic enzymes can modify intracellular compounds before interacting with parasite proteins, which are significant limitations of intracellular overexpression screens. However, the surface expression of proteins facilitates ligand–target interactions with little, if any, interference from yeast metabolic enzymes. Furthermore, each yeast cell can express ∼10^5^ copies of a single protein on its surface, which provides multiple ligand interacting sites and facilitates eukaryotic post-translational modifications of expressed proteins, which are not present in prokaryotic systems such as phage display. The surface display approach can also be used for numerous applications, including ligand-target binding screens, e.g., antibody binding, or phenotypic screens, e.g., drug survival screens.

We performed a proof-of-concept drug screen with yeast expressing the *G. lamblia* library. Although most applications of YSD are ligand-binding, we reasoned that a YSD-FS is more appropriate for drug targets. The YSD-FS does not require drug modifications (e.g., drug labeling with fluorochromes or capturing tags) that can interfere with binding to the unknown target. We used metronidazole because the drug is extensively used to treat *G. lamblia*, *Trichomonas varginalis*, and bacterial infections.^33^ Moreover, data on a few metronidazole interacting proteins were also available to cross-validate the screen.^27,34^ The screen identified genes encoding proteins that interact with metronidazole, e.g., thioredoxin reductase and purine nucleoside phosphorylase, thus reproducing described drug targets, and identified new protein interacting candidates. The annotated candidate interacting proteins’ function was predominantly related to heterocyclic compound binding, with enrichment in proteins with nucleotide-binding domains. The data suggest that metronidazole and perhaps its derived molecules might interact with ATP-binding proteins such as the NEK kinases or enzymes containing nucleotide-binding domains. In agreement with this, metronidazole has been shown to interact with enzymes involved in DNA metabolism,^27,33,34^ many of which we identified on the screen. Several Protein 21.1, which are rich in Ankyrin domains and share similarities with the NEK kinases, were identified, likely due to reminiscent kinase/ATP binding domains.^35^ Hence, our data suggest that metronidazole might target multiple proteins sharing conserved nucleotide-binding domains since the enriched reads mapped to sequences covering nucleotide-binding domains rather than other protein segments, implying potential sites of protein–drug interactions. The number of enzymes with nucleotide-binding domains is significant in any organism. However, the identification of dozens of enzymes on the screen suggests that amino acid differences within the nucleotide-binding domains could affect drug binding and indicates an apparent preference for some nucleotide-binding sequences. Although further validation of the metronidazole interacting protein candidates will be required, the screen points out directions for this drug targets and mechanisms of action. It might also guide structure–activity relationship experiments to design new target-based compounds. The results also indicate the usefulness of the YSD-FS in identifying potential drug interactors at nucleotide resolution.

A potential YSD-FS pitfall is that yeast cells may not be sensitive to the studied drugs. For example, yeast is highly resistant to metronidazole, and the concentrations used in the study differ from those used against *G. lambliain vitro.* However, the identification of validated metronidazole targets in the screen suggests that the assay tolerates drug ranges different from the concentrations used against the target pathogen. Nevertheless, using mutant yeast strains in multidrug resistance genes may be an alternative in the YSD-FS. Alternating yeast growth conditions from aerobic to anaerobic may also be an alternative to expand the assay application. Notably, the libraries were designed to express polypeptides covering the length of protein domains^20−22^ and thus have the advantage of identifying potential drug-binding sites or antibody epitope targets. However, interactions that might require a full-length protein may be missed as well as proteins or domains not appropriately folded on the yeast surface. The method developed to generate the libraries combined with the YSD-FS and high-throughput sequencing will help expand drug target discovery and mechanisms of action to organisms in which genetic manipulations are challenging, and tools are not well developed.

## Methods

### Cell Culture

*S. cerevisiae* strain EBY100 was obtained from the American Type Culture Collection (6). Suspension cultures were grown at 30 °C with platform shaking at 225 rpm. Cultures were maintained at 30 °C in YPD medium (10 g/L yeast extract; 20 g/L peptone; 20 g/L dextrose; pH 6.5) or YPD/agar (YPD with 20 g/L agar). Transformed yeast cells were cultured in a synthetic-defined (SD) medium without tryptophan (SD/-trp broth, Takara Bio USA Inc.) and induced in SD/-trp broth with 2% raffinose as an alternative carbon source by the addition of 1 to 3% galactose. *T. brucei* single-marker Lister 427 strain bloodstream forms were grown in HMI-9 medium supplemented with 10% inactivated Fetal Bovine Serum (FBS, Life Technologies)^36^ and 2 μg/mL G418 (Biobasic) at 37 °C with 5% CO_2_. *T. cruzi* Sylvio X10 strain epimastigotes (wildtype) were grown in Liver Infusion Tryptose medium supplemented with 10% inactivated FBS (Life Technologies) at 27 °C.^37^*G. lamblia* WB isolate (wildtype) was maintained in a modified TY-S-33 medium supplemented with 10% inactivated FBS and 2% Diamond’s vitamin solution at 37 °C.^38^

### Generation of Genome-Wide Libraries

A detailed protocol for library construction is available.^26^ Briefly, genomic DNAs were obtained from 1.0 × 10^8^ parasites. Cells were resuspended in 200 μL of lysis buffer (10 mM Tris–HCl, pH 8, 10 mM EDTA, 1% sodium dodecyl sulfate) and incubated with 20 units of Proteinase K for 30 min at 55 °C. Afterward, 20 μL of RNAse A (10 mg/mL) was added, and the samples were incubated at 22 °C for 5 min. Lysates were mixed vigorously with 1:1 (v:v) Phenol:Chloroform:Isoamyl Alcohol (25:24:1, pH 6.7) followed by centrifugation at 14,000*g* for 10 min. The aqueous phase was mixed 1:1 (v:v) with cold isopropanol and 5 μg of linear polyacrylamide and centrifuged at 14,000*g* for 15 min to precipitate DNA. The DNA pellets were washed with 70% cold ethanol and resuspended in 10 mM Tris–HCl pH 8. Next, 2 μg of genomic DNA was fragmented using an ultrafocused sonicator (Covaris M220, Covaris, Inc.) in microTUBE-50 AFA Fiber Screw-Cap tubes. *G. lamblia* genomic DNA was sonicated with 25 W peak incident power, 2% duty factor, 200 cycles, for 20 s at 20 °C. *T. brucei* genomic DNA was sonicated with 25 W peak incident power, 10% duty factor, 200 cycles per burst, for 10 s at 20 °C. *T. cruzi* genomic DNA was sonicated with 25 W peak incident power, 10% duty factor, 200 cycles per burst, for 3 s at 20 °C. DNA fragments of 0.5–3 Kb were size selected using Mag-Bind TotalPure NGS (Omega Bio-Tek) with a beads:sample ratio of 0.5× following the manufacturer’s instructions. DNA fragments were end-repaired and A-tailed using a NEBNext Ultra II End Repair/dA-Tailing Module (New England Biolabs Ltd.) according to the manufacturer’s instructions and then purified with a 0.5× beads:sample ratio as described above. The purified DNA was ligated to forked barcode adapters (Oxford Nanopore Technologies) using the Blunt TA ligase master mix (New England Biolabs Ltd.) for 2 h at 20 °C. The reaction product was purified using a 0.5× beads:sample ratio with Mag-Bind TotalPure NGS (Omega Bio-Tek), as described above. Then, 20 ng of DNA was used for PCR amplification using forward 5′-CGATGACGATAAGGTACCAGGATCCTTTCTGTTGGTGCTGATATTGC-3′ and reverse 5′-TGCAGAATTCCACCACACTGGATTACTTGCCTGTCGCTCTATCTTC-3′ primers. PCR was performed using Taq DNA Polymerase with ThermoPol Buffer (New England Biolabs Ltd.) with denaturation at 95 °C for 3 min followed by 15 cycles of 95 °C for 30 s, 49 °C for 30 s, and 72 °C for 3.5 min. PCR products were size selected with 0.5× beads:sample ratio with Mag-Bind TotalPure NGS (Omega Bio-Tek). Then, 47.04 fmol of fragmented DNAs was used for Gibson assembly with 25.83 fmol of BamHI-digested pYD1 vectors using NEBuilder HiFi DNA Assembly Master Mix (New England Biolabs Ltd.), and reactions were incubated for 1 h at 50 °C. One μL of the reaction mix was used to transform 50 μL of chemically competent (∼10^9^ cfu/μg) DH5α *Escherichia coli*. Ampicillin-resistant colonies were scraped from plates and combined, and the plasmid DNAs were isolated using the NucleoBond Xtra Midi EF plasmid mid-prep kit (Takara Bio USA Inc.) according to the manufacturer’s instructions.

### Nanopore Sequencing

Libraries cloned in the pYD1 vector were amplified using forward 5’-TTAAGCTTCTGCAGGCTAGTGGTG-3′ and reverse 5’-CACTGTTGTTATCAGATCAGCGGG-3′ primers with Taq DNA Polymerase and ThermoPol Buffer (New England Biolabs Ltd.) for 16 cycles of 95 °C for 30 s, 52 °C for 30 s, and 68 °C for 3.5 min. Amplified fragments were purified using 0.85x beads:sample ratio with Mag-Bind TotalPure NGS (Omega Bio-Tek). Fragments were prepared for Oxford nanopore sequencing using ligation sequencing kit SQK-LSK110 (Oxford Nanopore Technologies) and PCR Barcoding Expansion kit (EXP-PBC001) according to the manufacturer’s instructions, except that quick T4 ligase (New England Biolabs Ltd.) was used for the BCA ligation instead of the blunt TA enzyme/buffer mix (New England Biolabs Ltd.) recommended. Fifty fmol of pooled barcoded libraries were sequenced in a MinION using an R9.4.1 flow cell FLO-MIN106 (Oxford Nanopore Technologies) for 24 h following the manufacturer’s instructions. A total of 2–8 Gb of DNA were sequenced. All sequences are available in the Sequence Read Archive (SRA) with BioProject numbers PRJNA851424, PRJNA851089 and PRJNA851903.

### Computational Analysis

All scripts used in this manuscript are included in the Supporting Information. A detailed protocol for library sequencing analysis is also available.^26^ Nanopore sequencing fast5 data from sequenced libraries were basecalled with Guppy (Oxford Nanopore Technologies), according to the manufacturer’s instructions. Fastq sequences were mapped to *T. cruzi* Sylvio strain reference genome (release 57), or *T. brucei* 427–2018 genome (release 51), or *G. lamblia* WB (release 56) reference genome using minimap2.^39^ Mapping statistics and format conversions were obtained using Samtools.^40^ Coverage analysis was performed with DeepTools.^41^ Data were visualized using Integrative Genomics Viewer^42^ and circlize^43^ following the developer’s instructions. Enrichment analysis of genes was performed using the EdgeR tool.^44^ Reads were aligned to the *G. lamblia* WB isolate reference genome with minimap2, and read counts were obtained using featureCounts (from package Subread) and enrichment analysis performed with the EdgeR tool using a likelihood ratio test comparing metronidazole-treated vs vehicle-treated yeast (see scripts in the Supporting Information). Volcano plots were generated using ggplot2 package^45^ in RStudio. For correlation analysis, sequencing reads were aligned to the reference genome as indicated above, and read counts per gene were obtained using the featureCounts tool (from package Subread). The raw counts were analyzed using the package EdgeR to obtain normalized read counts per million. The Pearson coefficient of correlation was used to compare groups using RStudio (Posit). GO analysis was obtained using the GO Enrichment analysis in GiardiaDB.org.^46^ The Libframe tool was developed using Python version 3.8 (https://github.com/cestari-lab/Libframe-tool).

### Yeast Transfection and Galactose Induction

EBY100 cells were cultured in Petri dishes containing YPD agar and incubated at 30 °C for 72 h. Five to ten colonies were inoculated in 125 mL of YPD medium to an optical density (OD) of 0.02 (OD was measured using a spectrophotometer (VWR) at 600 nm wavelength) and incubated at 30 °C with shaking at 225 rpm overnight to reach an OD of 1.6. To produce electrocompetent yeast, the cells were harvested by centrifugation (2000*g*, 5 min, 4 °C) and pellets were resuspended in ice-cold water for cell washing and then centrifuged again at 2000*g* for 5 min at 4 °C. The procedure was repeated twice, and pellets were resuspended in ice-cold electroporation buffer (1 M sorbitol, 1 mM CaCl_2_). Cells were pelleted by centrifugation (2000*g*, 5 min, 4 °C) and resuspended in 25 mL of conditioning buffer (0.1 M LiAc/10 mM DTT) for 20 min at 30 °C with shaking at 225 rpm. Cells were harvested by centrifugation (2000*g*, 5 min, 4 °C) and washed once in ice-cold water and once in ice-cold electroporation buffer (as indicated above). Cells were harvested (2000*g*, 5 min, 4 °C) and resuspended in electroporation buffer to a final volume of 1.25 mL and kept on ice until electroporation. For each electroporation, 0.1 μg of library DNA and 5 μL (250 μg/mL final concentration) of salmon sperm DNA (ThermoFisher Scientific) were added to 200 μL of electrocompetent cells and mixed by gently pipetting. The DNA/yeast mixture was transferred to a pre-chilled BioRad GenePulser cuvette (0.2 cm electrode gap) (Bio-Rad) and incubated on ice for 5 min. Cells were then electroporated at 2.5 kV, 25 μF, and 200 Ω using a Bio-Rad Gene Pulser II electroporator (Bio-Rad). Cells were immediately transferred to a 4 mL mixture of 1:1 sorbitol:YPD and allowed to recover for 1 h at 30 °C with shaking at 225 rpm. Cells were collected by centrifugation at 2000*g* for 5 min at 4 °C, washed three times in water (as indicated above), and resuspended in 4 mL of SD/-trp. A 100 μL of the culture was taken for plating and calculation of transformation efficiency (see below). The remaining cells (3.9 mL) were added to 125 mL of SD/-trp and incubated for 24 h at 30 °C with shaking at 225 rpm. The culture was centrifuged (2000*g*, 5 min, 4 °C), and aliquots of ∼4.0 × 10^8^ transformed cells grown at a mid-log phase were frozen in SD/-trp media with 25% glycerol. For transformation efficiency, the 100 μL culture was 10-fold serial diluted and the 1:100 and 1:1000 dilutions were plated onto SD/-trp agar and grown at 30 °C. The transformation efficiency was calculated from the colony counts after 72 h. A mean transformation efficiency of 4.62 × 10^7^ ± 6.92 × 10^6^ CFU/μg of DNA (*n* = 15) was obtained (Table S1). To induce the library expression, one mL aliquot of transformed yeast was thawed and resuspended in 30 mL of SD/-trp/2% glucose and grown at 30 °C with shaking at 225 rpm to an OD of 1, measured at 600 nm wavelength using a spectrophotometer (VWR). Then, the cells were collected by centrifugation at 2000*g* for 5 min at 4 °C, washed three times with cold sterile water (as indicated above), and resuspended in 30 mL of SD/-trp with 2% raffinose (raf) to an OD of 1 (∼1.6 × 10^7^ cells/mL). The cells were incubated for 4 h at 30 °C with shaking at 225 rpm, and then, 8 mL of the culture (corresponding to 1.3 × 10^8^ total cells) was collected by centrifugation (2000*g*, 5 min, 4 °C) and resuspended in 10 mL (1.3 × 10^7^ cells/mL, OD 0.8) of SD/-trp/2% raf with the addition of 1–3% galactose to induce protein expression for 16 h at 30 °C with rotation at 225 rpm.

### Western Blotting

Protein extracts were prepared from 10 mL of yeast cells growing at an OD of 2 measured at 600 nm wavelength with a spectrophotometer (VWR). Cells were harvested at 2000*g*, and pellets were resuspended in 200 μL of 0.1 M NaOH and incubated at 22 °C for 5 min. Then, cells were centrifuged at 2000*g*, and then pellets were resuspended in 200 μL of lysis buffer, composed of 250 mM Tris (pH 6.8), 140 mM sodium dodecyl sulfate (SDS), 30 mM bromophenol blue, 27 μM glycerol, and 0.1 mM dithiothreitol, as previously described.^47^ Afterward, 200 μL of lysate was incubated at 95 °C for 10 min, the samples were centrifuged for 5 min at 5000*g* to pellet cell debris. Solubilized proteins (20 μL of lysate, corresponding to 12.5 μg of proteins) were resolved in 12% SDS/PAGE and transferred to PVDF membranes as previously described.^48^ Membranes were blocked with 6% non-fat dry milk in PBS 0.05% Tween (PBS-T) for 1 h at 22 °C. Afterward, membranes were incubated in mouse anti-Xpress antibodies (Invitrogen) diluted 1:1000 in blocking buffer (6% non-fat dry milk in PBS-T) for 1 h at 22 °C and then washed five times at 22 °C in PBS-T for 10 min each wash. The membranes were incubated in goat anti-mouse IgG-HRP (Santa Cruz) diluted 1:5000 in blocking buffer for 1 h at 22 °C, washed five times for 10 min each wash in PBS-T at 22 °C, and then developed with ECL chemiluminescence solution (Life Technologies) in a ChemiDoc Imaging System (Bio-Rad). Membranes were stripped by washing for 30 min in 100 mM Glycine pH 2.9, washed twice in water for 15 min each wash, and re-probed, as described above, with rabbit anti-β-actin (1:2000) (ABclonal Technology) and donkey anti-rabbit IgG 1:5000 (Santa Cruz). Membranes were developed as described above.

### Fluorescence-Based Imaging and Flow Cytometry Analysis

One mL aliquots containing approximately 2.0 × 10^7^ yeast cells were harvested by centrifugation (2000*g*, 5 min) and washed once in Milli-Q water and once in PBS. Cells were then resuspended in 1 mL of 1% paraformaldehyde (Thermo Scientific) diluted in PBS for 10 min at 22 °C with rotation. Glycine (180 mM) was added and rotated for an additional 10 min to halt fixation. Cells were washed three times in FACS buffer (PBS, 1% BSA, 0.1% sodium azide) and resuspended in 1 mL of FACS buffer. Cells (100 μL) were transferred to clean Eppendorf tubes (Ultident Scientific), pelleted as above, and resuspended in 100 μL of FACS buffer containing mouse anti-Xpress antibodies (Invitrogen) diluted 1:500 and incubated at 22 °C for 1 h with rotation. Cells were then washed three times in FACS buffer and incubated with AlexaFluor 488 conjugated goat anti-mouse IgG (Invitrogen) in FACS buffer for 1 h at 22 °C in the dark with rotation. Cells were washed three times in FACS buffer (as above) and then resuspended in 1 mL of FACS buffer for analysis. Twenty-five thousand cells were analyzed using the Attune NxT Flow Cytometer (ThermoFisher Scientific). For Cytation 5 imaging analysis and microscopy, cells were fixed and incubated with antibodies, as described above. Imaging analysis was performed using a Cytation 5 imaging reader (BioTek) with 20× magnification (Plan, Fluar, Phase objective) and 0.45 numerical aperture using Gen5 software (BioTek). For microscopy, yeast cells (treated as described above) were added to cover glass pre-soaked with 1% poly-l-lysine. Cells were mounted on slides with Fluoromount-G Mounting Medium (Thermo Scientific). The slides were analyzed using a Nikon E800 Upright fluorescence microscope (Nikon) with Nikon microscope objective Plan Apo 60XA/1.40 oil differential interference contrast (DIC) H ∞/.17 working distance (WD) 0.21 using NIS-Elements Imaging Software (Nikon).

### YSD-FS Screen of Metronidazole

Gl-Lib or pYD1 transformed yeast were inoculated into SD/-trp/2% raf/3% gal and incubated at 30 °C, 225 rpm for 16 h. The cultures were diluted to obtain an OD of 0.2, measured at 600 nm wavelength using a spectrophotometer (VWR), then 200 μL was inoculated on sterile Petri dish plates (150 mm × 25 mm) containing SD/-trp/2% raf/3% gal/agar medium and metronidazole (20 mg/mL, diluted in dimethyl sulfoxide, DMSO). Cultures were also incubated in plates with a drug vehicle (DMSO) only to monitor the effects on cells. After a 3 day incubation at 30 °C, the colonies from Gl-Lib or pYD1 transformed yeast grown in the presence of metronidazole were scraped from the plates. The cultures were recovered in SD/-trp medium for 16 h at 30 °C and then harvested by centrifugation (2000*g*, 4 min, 4 ° C), and pellets were resuspended in Milli-Q water to wash the cells; the procedure was repeated three times. Then, cells were replated as described above in metronidazole-containing plates for additional three rounds of selection (i.e., a total of four rounds of selection). Afterward, colonies were scraped from the plates, and their plasmid DNAs were extracted by resuspending the colonies in 400 μL of lysis buffer (10 mM Tris (pH 8.0), 0.1 M EDTA, 0.5% SDS) and 200 μL of acid-washed 0.5 mm glass beads. The mixture was incubated with 20 units of Proteinase K (New England Biolabs Ltd.) for 30 min at 55 °C. Then, it was vigorously vortexed for 10 min, with the subsequent addition of another 200 μL of lysis buffer and vortexed for 10 additional minutes. Afterward, 20 μL of RNAse A (10 mg/mL) was added to the mixture and incubated at 22 °C for 5 min. The mixture was boiled at 100 °C for 3 min in a heat block, placed on ice for 1 min, and then centrifuged for 14,000*g* for 10 min. The supernatant was collected for plasmid DNA extraction using 1:1 (v:v) Phenol:Chloroform:Isoamyl Alcohol (25:24:1) pH 6.7 followed by centrifugation at 14,000*g* for 10 min. Cold isopropanol 1:1 (vol:vol) and 3 μL (15 μg) of linear polyacrylamide were mixed with the aqueous phase and centrifuged at 14,000*g* for 15 min. The precipitated plasmid DNA was washed in 1 mL of 70% cold ethanol and resuspended in 100 μL of 10 mM Tris pH 8.0. The screen was performed with three biological replicates and samples multiplexed for Oxford nanopore sequencing as described above.

### Data and Statistical Analysis

Data are shown as means ± standard deviation of the mean from at least three biological replicates. Comparisons among groups were made by a two-tailed *t*-test using GraphPad Prism. *P*-values ≤0.05 with a confidence interval of 95% were considered statistically significant unless otherwise stated. Graphs were prepared using Prism (GraphPad Software, Inc.), Matlab (Mathworks), Integrated Genome Viewer (Broad Institute), or RStudio (Posit).

## Supporting Information Available

Complete list of genes enriched or depleted after YSD-FS screen with metronidazole. Dataset includes gene ID, log FC, log CPM, *F* and *p-*values, and product name (XLSX)
Enrichment of reads aligning to large repetitive gene families, flow cytometry analysis of non-induced or induced library, correlation of drug-treated versus non-transformed library, *G. lamblia* library transformation in *S. cerevisiae* EBY100, and scripts for computational data analysis (PDF)

## Notes

All data have been included within the article and Supporting Information. Sequencing data were deposited in the Sequence Read Archive (SRA) with BioProject numbers PRJNA851089, PRJNA851424, and PRJNA851903. The Libframe tool and its code is available at https://github.com/cestari-lab/Libframe-tool. The genome-wide libraries are available upon request (Contact: Igor Cestari, igor.cestari@mcgill.ca).
