# Supplementary material for: Genome-Wide Libraries for Protozoan Pathogen Drug Target Screening Using Yeast Surface Display
Source: ACS Infect Dis. 2023 Apr 21;9(5):1078–91. doi: 10.1021/acsinfecdis.2c00568 (PMC10187560; doi:10.1021/acsinfecdis.2c00568)
Supplement: Supplementary file 2 — id2c00568_si_002.pdf [file id2c00568_si_002.pdf]

Supporting Information

Genome-wide libraries for protozoan pathogens drug target screening using yeast surface display

Rhiannon Heslop[1,3], Mengjin Gao[1], Andressa Brito Lira[1], Tamara Sternlieb[1], Mira Loock[1], Sahil Rao Sanghi[1], and Igor Cestari[1,2,*]

[1] Institute of Parasitology, McGill University, Ste Anne de Bellevue, QC H9X 3V9, Canada

[2] Division of Experimental Medicine, McGill University, Montreal, QC, H4A 3J1, Canada

[3] Faculté de Pharmacie de Tours, 31, Avenue Monge, 37200, Tours, France

*Correspondence: igor.cestari@mcgill.ca

This file contains:

- Figures S1-S3
- Table S1
- The scripts used for computational analysis in this work.

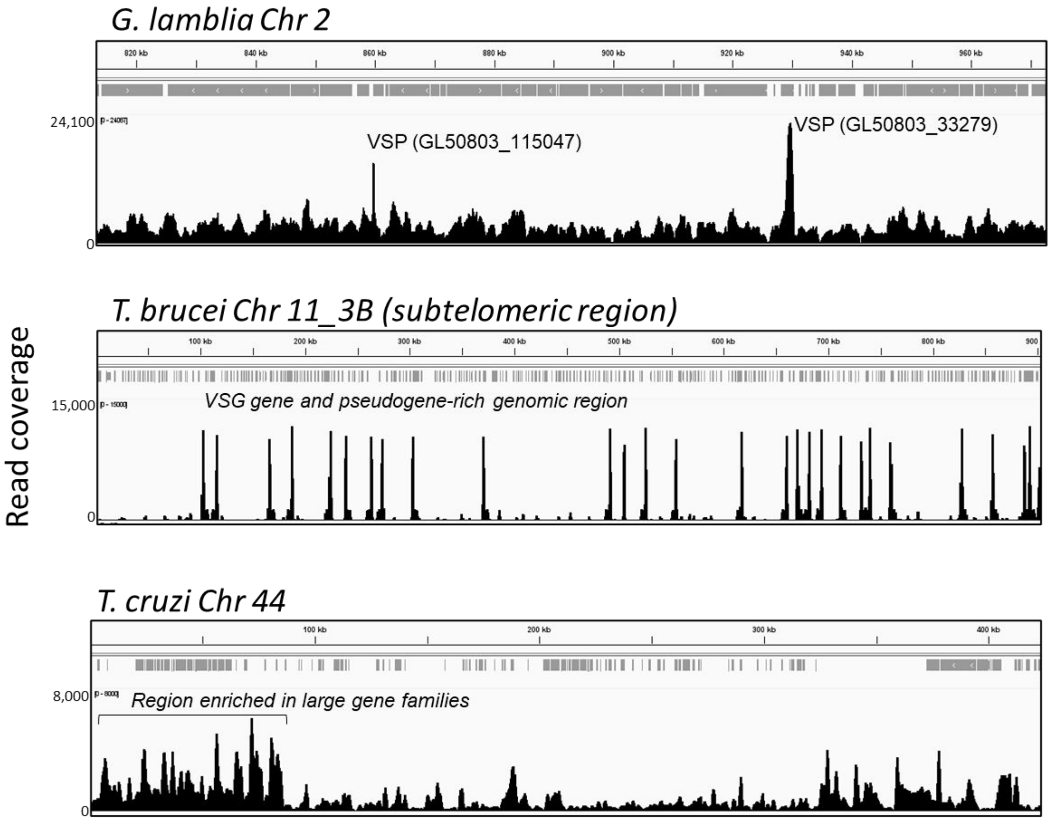

Figure S1. Enrichment of reads aligning to large repetitive gene families. Top, read coverage (in black) over a segment of *G. lamblia* chromosome (Chr) 2. Overly enriched sequences (read coverage peaks) map to variant surface proteins (VSPs). Middle, read coverage (in black) over a segment of *T. brucei* subtelomeric region containing variant surface glycoprotein genes (VSGs) and pseudogenes. Bottom, read coverage (in black) over a segment *T. cruzi* Chr 44 showing enriched sequences mapping to the subtelomeric region containing dispersed repetitive gene families. Genes are indicated by gray bars/rectangles.

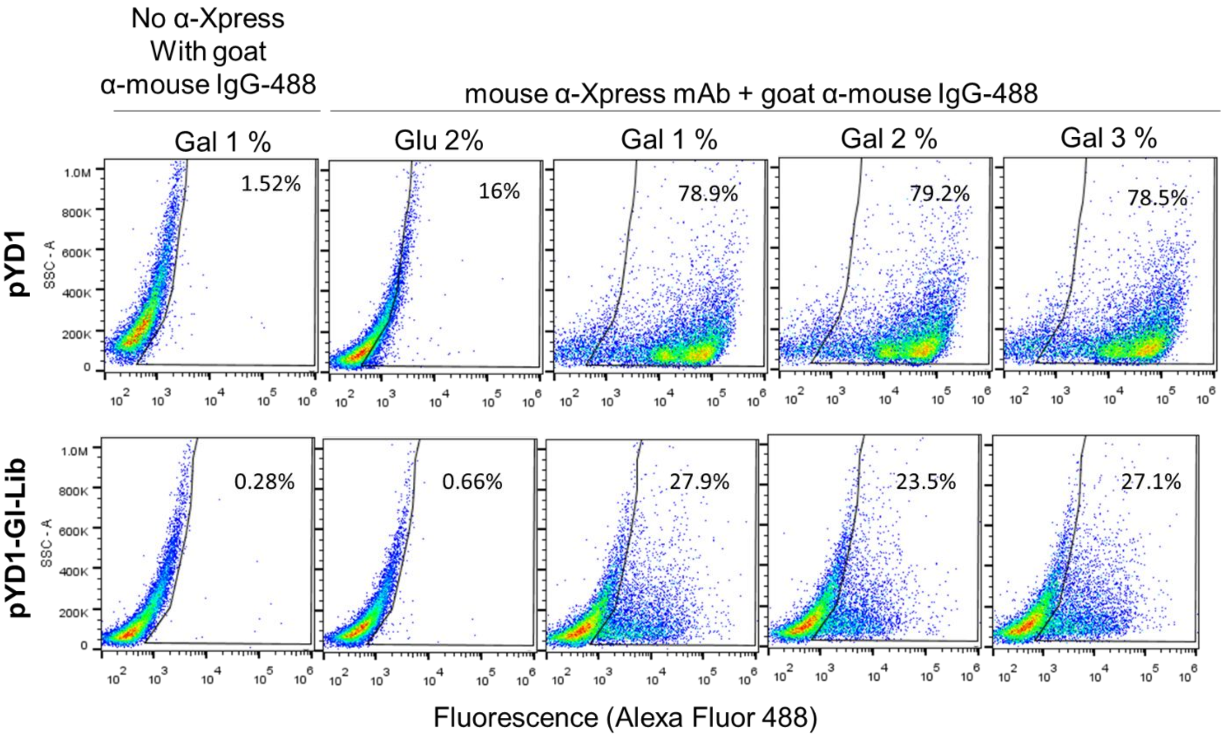

Figure S2. Flow cytometry analysis of non-induced or induced library. Plots show the yeast cells transformed with pYD1 or Gl-lib (i.e., pYD1-*G. lamblia* library) stained or not with monoclonal antibodies against the Xpress epitope. Yeast cultures were grown in a medium containing 1, 2, or 3% galactose to induce library expression or non-induced (repressed) with 2% glucose (Glu). The percentage (%) of positive populations is shown in the graph. SSC-A, side scatter parameter.

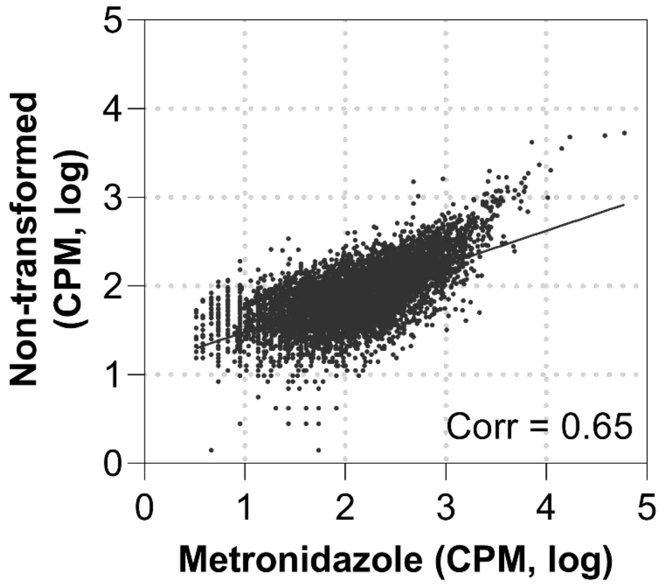

Figure S3. Correlation of drug-treated versus non-transformed library. Scatter plot of reads counts per million (CPM) of *G. lamblia* non-transformed library compared to yeast expressing Gl-Lib grown in the presence of metronidazole. The Pearson coefficient of correlation (Corr) comparing datasets is shown. The line shows linear regression comparing both libraries. Reads were aligned to the genome using the minimap2 tool, and read counts per gene were obtained using the featureCounts tool (from package Subread). The raw counts were analyzed using the package EdgeR to obtain normalized read counts per million, and Pearson's coefficient of correlation was calculated using RStudio (Posit). The graph was prepared using GraphPad Prism (GraphPad Software Inc). Scripts used for analysis are available below. See also Sternlieb et al. (26) protocol for additional information on computational analysis.

Table S1. *Giardia lamblia* library transformation in *Saccharomyces cerevisiae* EBY100. Fifteen transformations were performed with *Gl-lib* by electroporation in three experimental groups (five transformations per group). Each transformation was performed with 100 ng of library DNA. [a]Calculated clones obtained by combining all 15 transformations. [b]Library size represents the number of clones obtained after transforming *Escherichia coli* with Gibson-assembled genomic fragments in pYD1. [c]Fold-change comparing the number of clones (from yeast transfection) vs library size. [d] Mean and SDM of Exp. groups mean 1,2, and 3. Exp., Experimental. SDM, standard deviation of the mean.

Exp. groups	Transformation Efficiency (cfu/µg)	MEAN and SDM	Number of clones [a]	Library size [b]	Fold-Change [c]
1	$3.32x10^7$	$3.27x10^7$ ± $4.03x10^6$			
	$3.31x10^7$				
	$2.66x10^7$				
	$3.78x10^7$				
	$3.37x10^7$				
2	$2.46x10^7$	$2.78x10^7$ ± $2.72x10^6$			
	$2.81x10^7$				
	$2.54x10^7$				
	$3.04x10^7$				
	$3.04x10^7$				
3	$5.42x10^7$	$7.80x10^7$ ± $2.39x10^6$			
	$5.85x10^7$				
	$8.42x10^7$				
	$1.14x10^8$				
	$7.92x10^7$				
-	-	[d] $4.62x10^7$ ± $6.92x10^6$	69,200,000	190,160	363.9

Scripts for computational data analysis

The scripts below were submitted to a cluster from Compute Canada (www.computecanada.ca/) using Linux Ubuntu in Windows Subsystem for Linux. A general file name is given for simplicity, e.g., "GenomeOfReference.fasta" for reference genome, or "reads-map_v1.sam" for mapped output file.

1. Decompressing fastq files and mapping the reads to the genome using minimap2.

```
#Before mapping, files in the *.fasta.tar.gz format must be decompressed
using the bash command

tar xvzf yourdata.fastq.tar.gz -C pathdirectory


#This script aligns the reads obtained from the ONT sequencing to the genome
of reference, creating a sam file.

module load minimap2/2.24


minimap2 -ax map-ont -k11 -m30 -w7 -I4G -t16 -2 GenomeOfReference.fasta \


/~/data/fastq/*.fastq.gz \


>/~/data/analysis/reads-map_v1.sam
```

2. Obtaining mapping statistics and files conversion from .sam to .bam files. Sorting and indexing .bam files.

```
#Samtools commands extract statistics from the read mapping and will create
the bam binary files for further processing.


module load samtools


samtools flagstat /~/data/analysis/reads-map_v1.sam > /~/data/analysis/reads-
map_v1-flagstat.txt


samtools stat /~/data/analysis/reads-map_v1.sam > /~/data/analysis/reads-
map_v1-stat_lib.txt


samtools view -S -b /~/data/analysis/reads-map_v1.sam >
/~/data/analysis/reads-map_v1.bam
```

```
samtools sort /~/data/analysis/reads-map_v1.bam > /~/data/analysis/reads-map_v1_sorted.bam
```

```
samtools index /~/data/analysis/reads-map_v1_sorted.bam > /~/data/analysis/reads-map_v1_sorted.bam.bai
```

3. Coverage analysis of the genome and read counting.

```
#Using the DeepTools module to perform further analysis and generate visualizations.

module load python/3.8.2

#For these steps a virutal environment was created

source ~/ENV/bin/activate


#plotCoverage generates a curve plot depicting the reads per base and the mean coverage of the genome.

plotCoverage -b /~/data/analysis/reads-map_v1_sorted.bam --labels reads-map_v1 --plotFileFormat pdf --outRawCounts Reads-V1_Lib.txt --numberOfProcessors 8


#Load required packages for readcount analysis (package subread). Read count was used to quantify the number of reads per gene.


module load nixpkgs/16.09

module load gcc/7.3.0

module load StdEnv/2020

module load subread/2.0.3


#featureCounts counts mapped reads for genomic features such as genes, exons, promoter, gene bodies, genomic binds and chromosomal locations. The script below will generate the number of read counts for each exon.

featureCounts -LMO -a GenomeOfReference.gtf -o reads-map_v1_counts.txt -F "exon" -g "gene_id" -s 0 -T4 reads-map_v1_sorted.bam
```

4. Processing the data for visualization of library coverage.

```
#bamCoverage generates a coverage track by normalizing and binning the reads, producing a *.bw file. The file can be analyzed in a genome visualization
```

```
tool (e.g., integrated genome viewer such as https://igv.org/app/) for visual validation and to check for potential bias in library coverage.

bamCoverage -b reads-map_v1_sorted.bam -o reads-map_v1.bw --binSize 20 --normalizeUsing RPKM --extendReads 1000 --outFileFormat bigwig --numberOfProcessors 10


#computeMatrix creates a matrix of scores per region that is necessary as intermediate for the visualization of the data as a heatmap.

computeMatrix scale-regions -b 500 -a 500 -m 3000 -R GenomeOfReference.gtf -S reads-map_v1.bw --skipZeros --sortRegions no --transcriptID 'exon' -o matrix_reads-map_v1-sr1.gz


#plotHeatmap generates the heatmap to visualize gene coverage using the matrix file from computeMatrix.

plotHeatmap -m  matrix_reads-map_v1-sr1.gz  --outFileName reads-map_v1_heatmap.png --colorMap RdBu --whatToShow 'heatmap and colorbar' --zMin -4 --zMax 4
```

5. Generate graphs for visualization of read coverage using circular plot in R.

```
#Circlize package was used to generate the circular visualization of genome coverage. First, load R.

module load r


#Initiate R

R


#Install required packages

install.packages("circlize")


#Loading necessary libraries

library(dplyr)

library(circlize)


#Reading data from the plotCoverage output file (reads per base)

Lib_Cov.df = read.table('Reads-V1_Lib.txt', sep = "\t")
```

```
#Order the table according to read counts

Lib_Cov.ordered<-Lib_Cov.df[order(Lib_Cov.df$V4), ]


#Filter the data frame to have only the reads from complete chromosomes. This
is a useful step in the case of partially sequenced or partially assembled
genomes of reference.

Lib_Cov.chrom <- dplyr::filter(Lib_Cov.ordered, grepl('CHR', V1)) %>%

#Converting the scale to log2 to improve visualization (optional)

  dplyr::mutate(V4 = log2(V4)) %>%

# Converting  all cells that became -inf during log conversion to NA.

  dplyr::mutate_if(is.numeric, list(~na_if(., -Inf)))


#Generate the circlize graph

#This first row inicializes the circular graph

circos.initializeWithIdeogram(Lib_Cov.chrom, plotType = NULL,
circos.par(gap.degree = 8))


#This chunk generates the most outer ring with the chromosomes tracks

circos.track(ylim = c(0, 1), panel.fun = function(x, y) {

  chr = CELL_META$sector.index #Uses the chromosome names as labels

  xlim = CELL_META$xlim

  ylim = CELL_META$ylim

  circos.rect(xlim[1], 0, xlim[2], 1, col = "lightblue") #Changes the color
of the chromosome rectangles

  circos.text(mean(xlim), mean(ylim), chr, cex = 1.5, col = "white",

              facing = "bending.inside", niceFacing = TRUE) #Changes the
color of the text and the way it is oriented inside the rectangles.

}, track.height = 0.15, bg.border = NA)


#This chunk adds the counts track

circos.genomicTrack(Lib_Cov.chrom,

                    panel.fun = function(region, value, ...) {

                      circos.genomicPoints(region, value, type = "segment",
lwd = 2,

                                           col = "magenta", cex = 0.5, ...)
```

```
                    circos.yaxis("right", labels.cex = 0.4, col = "grey",
labels.col = "darkgrey")
                    })
#This adds a text in the middle of the circle
text(0, 0, "Genome\ncoverage", cex = 1.5)
```

6. Libframe analysis to calculate the predicted peptide lengths and amino acid sequences.

Note. The Libframe tool used in this step was developed in python and is used for pYD1. If using a different expression system, the code can be modified to replace the Xpress tag sequence with any sequence that should be in-frame with the cloned fragments. The tool with code assessable is available at https://github.com/cestari-lab/Libframe-tool. To access the code, open the file using a text editor.

```
#The libframe script finds the Xpress tag sequence in the reads and
translates everyhting in frame after the end of the tag and until a STOP
codon is found.

#Load python and activate enviroment
module load python/3.8.2
source ~/ENV/bin/activate

#Install biopython
pip install biopython

#Decompress the *.fasta.gz files into *.fasta files and concatenate all the
multiple FASTA files.
gunzip *.fastq.gz
cat *.fastq > newfilename.fastq

#Execute libframe. It will output a text file with peptides' length and
sequence. It takes three commands: 1) path to fastq file; 2) path to output
file (.txt); and 3) the minimum length of a peptide. The Xpress tag has 8 aa
(24 bases), plus linker (6 bases, 2 aa), and restriction site (Bam HI, 6
bases, 2 aa) resulting in 12 aa sequence DLYDDDDKVPGS. Hence, we recommend a
minimum value of 13 for a peptide.

python3 ./libframe.py path/to/fastq/file/.fastq path/to/output/file/.txt 13
```

```
#Example of results:

#Protein length is 127 aa and sequence is:
DLYDDDDKVPGSTSHAPGRHGGRRIRLELHLDNFKLLPQLCSSVSADGSPAVPLQPVLPGIRQMLHHSVSIKCADAR
VARFLWPCPYALHCALLSITSEFAAACGSTIWISVVEFCEISSTVAAARV

#Protein length is 16 aa and sequence is: DLYDDDDKVPGSLCWC

#Protein length is 75 aa and sequence is:
DLYDDDDKVPGSFLLVLILRRLLGCLTLIRAERHNRPQQGFVRRTAGVFHPSVATKSTCVFHFCISMYGRICGLL

#Protein length is 25 aa and sequence is: DLYDDDDKVPGSFVGADIAGDRAGK

#Protein length is 73 aa and sequence is:
DLYDDDDKVPGSFLLVLILRRLHGCLTLCLRVHTLDTKQPEYPLLGGISRAPAHKGAALGGSFSSGCLHAEEV
```

7. Statistical analysis of metronidazole treated vs non-treated samples using edgeR.

```
#Circlize package was used to generate the circular visualization of genome
coverage. First, load R.

module load r


#Initiate R

R


#Load required libraries

library (limma)

library (edgeR)


#Create DGEList and statistical design

y <- DGEList(counts=data)

group <- factor(c(1,1,1,2,2,2))

design <- model.matrix(~group)

y <- calcNormFactors(y)


#Generate plot MSD for sample comparisons

plotMDS.pdf <-plotMDS(y, col=c(rep("black",2), rep("red",2)))
```

```r
#Estimate sample dispersion

y <- estimateDisp(y, design)


#Plot Biological correlate of variation

plotBCV(y)


#Generate statistical analysis using generalized linear model

fit <- glmQLFit(y, design)


# Compare groups treated vs non-treated

qlf.2vs1 <- glmQLFTest(fit, coef=2)
```
